# Simultaneous Improvement in the Strength and Formability of Commercially Pure Titanium via Twinning-induced Crystallographic Texture Control

**DOI:** 10.1038/s41598-019-38652-1

**Published:** 2019-02-14

**Authors:** Jong Woo Won, Chan Hee Park, Jaekeun Hong, Chong Soo Lee, Seong-Gu Hong

**Affiliations:** 1 Metal Materials Division, Korea Institute of Materials Science, Changwon, 51508 Republic of Korea; 2 Graduate Institute of Ferrous Technology, Pohang University of Science and Technology, Pohang, 37673 Republic of Korea; 3 Division of Industrial Metrology, Korea Research Institute of Standards and Science, Daejeon, 34113 Republic of Korea; 4 Department of Nano Science, University of Science and Technology, Daejeon, 34113 Republic of Korea

## Abstract

The rolling texture formed in the conventional cold rolling process of commercially pure titanium (CP-Ti) for producing a metal sheet significantly limits the potential applications of CP-Ti sheets in various industrial sectors by impairing the formability. Here, we report that by exploiting a twinning-induced crystallographic texture modification, the rolling texture can be weakened and dispersed effectively, leading to a simultaneous improvement in the formability and yield strength. A two-stage cold rolling process was designed with intermediate annealing at a late stage of the conventional cold rolling process to generate deformation twins. The intermediate annealing drove the activation of $\{11\bar{2}2\}$ twin and $\{11\bar{2}2\}$ – $\{10\bar{1}2\}$ double twin in the second stage of the rolling process by removing the internal reaction stress developed in the first stage of the rolling process through recrystallization, and the crystallographic feature of the $\{11\bar{2}2\}$ twinned region, i.e., $\{11\bar{2}2\}$ twin texture, was effective for <*a*> type slips and $\{10\bar{1}2\}$ twinning to accommodate a through-thickness strain as well as for reducing the planar anisotropy. This enhanced thinning capability and reduced planar anisotropy in the $\{11\bar{2}2\}$ twin texture led to an improvement of the formability. We demonstrated the feasibility of the suggested two-stage cold rolling process with ASTM grade 2 CP-Ti.

## Introduction

Because of the superior corrosion resistance, high specific strength, good weldability, and biocompatibility, commercially pure titanium (CP-Ti) is now being widely used in various industrial fields including power generation, water desalinization, petrochemical, chemical, and biomedical industries^1^. One of its major applications is the manufacture of heat exchangers, in which it is used in a form of a thin sheet with complex wave patterns to enhance the heat transfer efficiency. Therefore, it is essential that it has a good stretch formability^1,2^. However, the rolling texture formed in the conventional cold rolling (CCR) process for producing a CP-Ti sheet has a specific crystallographic feature with most of the *c*-axes of the hexagonal close-packed (HCP) lattice inclined at ±(25°–35°) from the normal direction (ND) toward the transverse direction (TD), i.e., a TD split basal texture^3–5^, and this increases the difficulty for <*a*> type slips to accommodate a through-thickness strain, reducing the thinning capability and consequently leading to a poor formability^6–9^. Thus, generally, high-purity CP-Ti sheets with a good deformation capability are used^2^. High-purity CP-Ti sheets, however, have an inferior mechanical strength owing to the low content of impurities, requiring the use of thicker sheets. Moreover, high-purity CP-Ti sheets have a higher manufacturing cost. It is known that the mechanical strength of CP-Ti sheets markedly improves with the increasing content of the impurities (e.g., oxygen and iron)^10–12^. Therefore, if the formability of low-purity CP-Ti sheets could be improved by the rolling process, then desirable CP-Ti sheets having a combination of high strength and superior formability could be achieved. This would strengthen the competitiveness of CP-Ti sheets not only by allowing the use of thinner sheets owing to the enhanced mechanical strength, thereby saving Ti raw material and improving the heat transfer efficiency, but also by lowering the manufacturing cost.

Recently, a crystallographic texture control using deformation twins was found to be effective for improving the formability of rolled Mg alloys^8,13–15^. The formation of deformation twins can induce a rapid and large-scale modification of the crystallographic orientation, thereby weakening and dispersing the rolling texture effectively^16,17^. However, the most important issue that should be solved for its application in the sheet manufacturing process is finding a method for generating deformation twins during the rolling process. We have noted that the typical rolling texture (the TD split basal texture) already develops in a late stage of the CCR process for producing a CP-Ti sheet^3–5^, with a crystallographic orientation favorable for the activation of the $\{11\bar{2}2\}$ twin and $\{11\bar{2}2\}$–$\{10\bar{1}2\}$ double twin during the remaining CCR process^18–20^. The CP-Ti material is under a compressive loading condition along the ND during the CCR. However, despite the favorable crystallographic lattice orientation for deformation twins, the twinning activity is thoroughly suppressed at this late stage of the CCR because of an increase in the internal reaction stress. The latter is a result of the grain refinement realized through the formation of elongated grain structures and twin boundaries as well as the increase in dislocations. Because the resolved shear stress required for activating a deformation twin (i.e., twinning stress) is a combination of the single-crystal critical-resolved shear stress and internal reaction stress, accounting for the current state of hardening^21^, the twinning stress increases with the increasing thickness reduction during the CCR, making the activation of the deformation twins difficult. The only approach for restoring the twinning activity is the removal of the microstructural factors generating the internal reaction stresses, and this can be realized by recrystallization.

In this study, we developed a two-stage cold rolling (TCR) process with intermediate annealing at a late stage of the CCR, which enabled the activation of deformation twins in the second stage of the rolling. This enablement was realized by removing the internal reaction stress generated in the first stage of the rolling process through recrystallization, thereby allowing an effective control of the crystallographic texture. The feasibility of the suggested TCR process was demonstrated with ASTM grade 2 CP-Ti, which has limited use owing to the low formability despite the ~60% higher yield strength than ASTM grade 1 CP-Ti. The underlying mechanisms for improved yield strength and formability were explored in terms of the twinning-induced crystallographic lattice reorientation and its effect on the activities of <*a*> type slips and $\{10\bar{1}2\}$ twinning. Moreover, an optimized TCR process was discussed based on the result.

## Results and Discussion

### Two-stage cold rolling process

**Figure 1:**
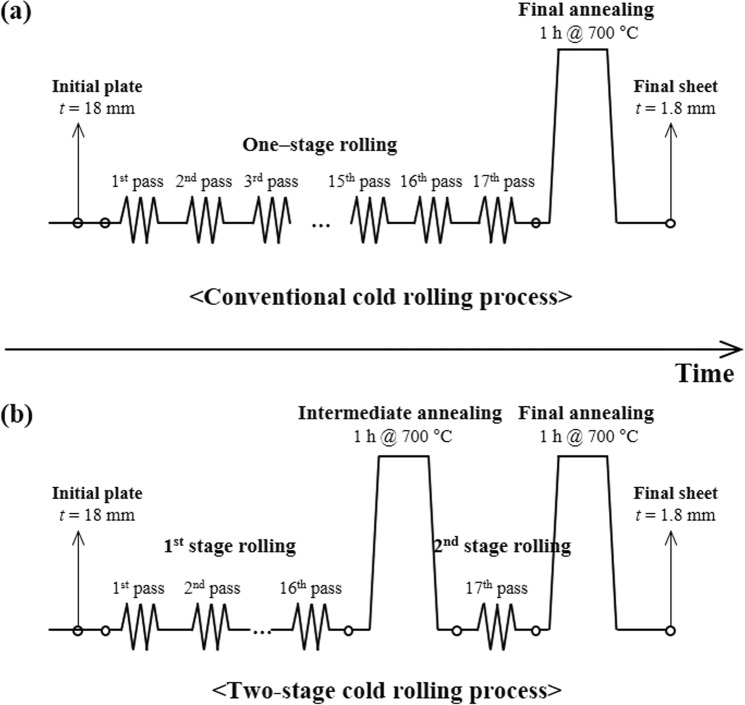
Schematics of (**a**) the CCR and (**b**) TCR processes. Both cold rolling processes comprise 17 rolling passes with a total thickness reduction of 16.2 mm.

A two-stage cold rolling process with intermediate annealing was designed considering the following factors. First, to produce a 1.8 mm thick CP-Ti sheet from an 18 mm thick CP-Ti plate, a total thickness reduction of 16.2 mm is imposed in the TCR, which is equal to that in the CCR process. Second, intermediate annealing is employed at a late stage of the CCR when the rolling texture is sufficiently developed so that the crystallographic lattice orientation is favorable for deformation twins. Third, $\{11\bar{2}2\}$ twin and $\{11\bar{2}2\}$–$\{10\bar{1}2\}$ double twin can form during the second stage of the rolling, and they have a completely different effect on the crystallographic lattice reorientation^19^. The amount of thickness reduction in the second stage of the rolling is varied, thereby controlling the activity of both the twin systems, so that the optimized condition for improving the yield strength and formability is explored. Figure 1 shows the schematics of the CCR and designed TCR processes. Both the rolling processes comprise 17 rolling passes with a total thickness reduction of 16.2 mm. In the CCR (Fig. 1a), a thickness reduction of 16 mm is imposed during the first 16 passes with a rate of 1 mm/pass, and a remaining 0.2 mm thickness reduction is imposed in the 17^th^ pass. In contrast, in the TCR (Fig. 1b), a thickness reduction of 15 mm is imposed during the first 15 passes with a rate of 1 mm/pass, and then the remaining 1.2 mm thickness reduction is imposed in the 16^th^ and 17^th^ passes. Intermediate annealing is employed after the 16^th^ pass at 700 °C for 1 h. Four different TCR processes are designed by varying the extent of the thickness reduction in the 17^th^ pass (i.e., the second stage of the rolling). Note that the percent thickness reduction in the second stage of the rolling varies from 10% to 36% (Table 1). A final recrystallization annealing at 700 °C for 1 h is employed in both the CCR and TCR processes.

**Table 1:** Thickness reduction in four designed TCR processes (unit: mm).

	Rolling pass	TCR #1	TCR #2	TCR #3	TCR #4
1^st^ stage rolling	1^st^ to 15^th^ passes	15	15	15	15
	16^th^ pass	1	0.8	0.5	0.2
2^nd^ stage rolling	17^th^ pass	0.2 (10%)*	0.4 (18%)	0.7 (28%)	1 (36%)

*Numbers in parentheses indicate the percent thickness reductions in the 17^th^ pass.

### Microstructural characteristics

**Figure 2:**
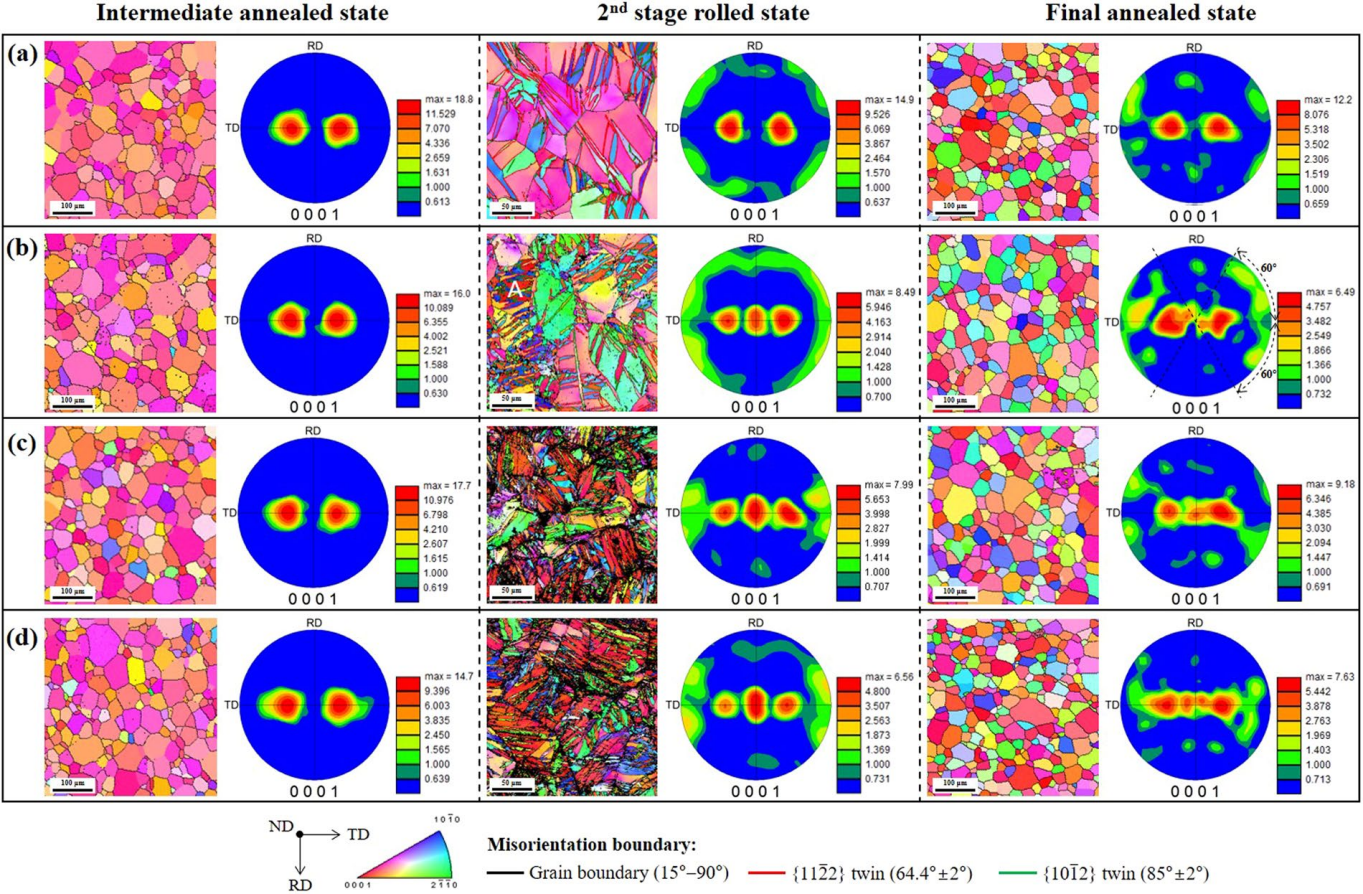
Inverse pole figure maps and (0001) pole figures showing the microstructural evolution during the TCR processes. (**a**) TCR #1, (**b**) TCR #2, (**c**) TCR #3, and (**d**) TCR #4.

The electron backscatter diffraction (EBSD) inverse pole figure maps and (0001) pole figures showing the microstructural evolution during the TCR processes are presented in Fig. 2; refer to the data of the CP-Ti sheet produced by the CCR process in Figure S1 in the Supplementary Information for comparison. In the intermediate annealed state, because the thickness reduction imposed in the first stage of the rolling is similar for all the four TCR processes (89% for TCR #1, 88% for TCR #2, 86% for TCR #3, and 84% for TCR #4), there is no difference in the microstructural characteristics. All the specimens are fully recrystallized and have an equiaxed grain structure with a similar grain size (36 μm for TCR #1, 40 μm for TCR #2, 34 μm for TCR #3, and 33 μm for TCR #4 on average) and a TD split basal texture with most of the *c*-axes inclined at ±(25°–35°) from the ND toward the TD. However, in the second stage of the rolled state, significantly different microstructures develop depending on the thickness reduction in the second stage of the rolling. In TCR #1, with a low thickness reduction of 10%, mostly $\{11\bar{2}2\}$ twins with a misorientation angle of 64.4° are observed and a new texture component with the *c*-axis distributed widely in the rolling direction (RD)–TD plane appears. When the thickness reduction is increased to 18% (TCR #2), profuse $\{11\bar{2}2\}$ twins are formed and some $\{11\bar{2}2\}$–$\{10\bar{1}2\}$ double twins with a misorientation angle of 85° are observed. The new texture component is intensified and another new texture component with the *c*-axis aligned parallel to the ND starts to develop. With further increase in the thickness reduction (28% for TCR #3 and 36% for TCR # 4), $\{11\bar{2}2\}$ twins and $\{11\bar{2}2\}$–$\{10\bar{1}2\}$ double twins are formed more profusely, and the texture component with the *c*-axis aligned parallel to the ND is intensified, whereas the texture component with the *c*-axis distributed widely in the RD–TD plane is weakened. It is noted that the TD split basal texture is gradually weakened with increasing thickness reduction. In the final annealed state, all the materials were fully recrystallized, leading to an equiaxed grain structure with similar average grain sizes of 34–39 μm. Interestingly, the main features of the crystallographic textures developed in the second stage of the rolling remain almost unchanged, indicating that the crystallographic lattice reorientation caused by the deformation twins is so stable that it can be maintained after recrystallization although the deformation twins disappear. These results clearly support that the suggested TCR process is capable of weakening and dispersing the TD split basal texture effectively by enabling the activation of the deformation twins.

**Figure 3:**
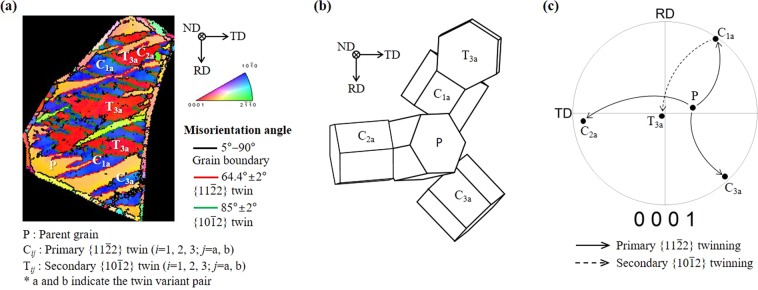
Crystallographic characterization of the parent grain and twins in grain A, indicated in the inverse pole figure map of the second stage of the rolled state in Fig. 2b. (**a**) Inverse pole figure map. (**b**) Crystallographic relationships between the parent grain, primary $\{11\bar{2}2\}$ twins, and secondary $\{10\bar{1}2\}$ twins. (**c**) (0001) pole figure.

As evidenced by the microstructural characteristics, the crystallographic texture evolution in the second stage of the rolling process arises from the formation of deformation twins. To understand the twinning characteristics and their role in the crystallographic lattice reorientation, crystallographic orientation analysis was conducted on the parent grain and twins in grain A, indicated in the inverse pole figure map of the second stage of the rolled state in Fig. 2b. As shown in Fig. 3, first, $\{11\bar{2}2\}$ twins are generated in the parent grain, i.e., there is primary $\{11\bar{2}2\}$ twinning, which is followed by $\{10\bar{1}2\}$ twin ($\{11\bar{2}2\}$–$\{10\bar{1}2\}$ double twins) appearance in the preformed $\{11\bar{2}2\}$ twins, i.e., there is secondary $\{10\bar{1}2\}$ twinning. It is known that for α-Ti, the $\{11\bar{2}2\}$ contraction twin and $\{10\bar{1}2\}$ extension twin are the most common twin systems at room temperature^19,22,23^, and their activation relies on which type of strain is developed along the *c*-axis of the HCP lattice^19,24^. $\{11\bar{2}2\}$ and $\{10\bar{1}2\}$ twins are activated when compressive and tensile strains are developed along the *c*-axis, respectively^19,24^. Considering the crystallographic orientation of the TD split basal texture in which the *c*-axis is inclined at ~30° from the ND toward the TD, the loading condition of the compression along the ND, which it experiences during the second stage of the rolling, introduces a compressive strain along the *c*-axis of the parent grain, and therefore, $\{11\bar{2}2\}$ twinning occurs. The crystallographic lattice rotation of 64.4° caused by $\{11\bar{2}2\}$ twinning makes the *c*-axis of the $\{11\bar{2}2\}$ twinned region almost parallel to the RD–TD plane (Fig. 3c). This crystallographic orientation undergoes a compression perpendicular to the *c*-axis during the remaining second stage of the rolling process, causing a tensile strain along the *c*-axis of the $\{11\bar{2}2\}$ twinned region, which is favorable for $\{10\bar{1}2\}$ twinning. Therefore, a $\{10\bar{1}2\}$ twin is formed in the preformed $\{11\bar{2}2\}$ twin. The $\{10\bar{1}2\}$ twinning induced crystallographic lattice rotation of 85° makes the *c*-axis of the $\{10\bar{1}2\}$ twinned region almost parallel to the ND (Fig. 3c).

Another important factor that affects the twinning-induced crystallographic texture modification is a variant selection mechanism during twinning. Because of the unique crystallography of the HCP lattice structure, there are six crystallographically equivalent variants in both the $\{11\bar{2}2\}$ and $\{10\bar{1}2\}$ twin systems, and thus, the variants that are active among these six variants are critical for modifying the crystallographic lattice orientation. To understand a variant selection mechanism during the primary $\{11\bar{2}2\}$ twinning and secondary $\{10\bar{1}2\}$ twinning, we conducted a Schmid factor (SF) analysis of the parent grain and twins in grain A (Table 2). For the convenience of describing the crystallographic orientation, Euler angles (*φ*_1_, *Φ*, *φ*_2_) with reference to the HCP crystal coordinates of the RD = $[1\bar{1}00]$, TD = $[2\bar{1}\bar{1}0]$, and ND = [0001] were used. C_ij_ and T_ij_ (i = 1, 2, 3; j = a, b; a and b indicate the twin variant pair) in Fig. 3 and Table 2 represent the six crystallographically equivalent $\{11\bar{2}2\}$ and $\{10\bar{1}2\}$ twin variants, respectively, and are defined as C_1a_ =$\,(\bar{1}2\bar{1}2)[1\bar{2}\mathrm{13}]$, C_1b_ = $(1\bar{2}{\rm{12}})[\bar{1}2\bar{1}3]$, C_2a_ = $({\rm{11}}\bar{2}2)[\bar{1}\bar{1}\mathrm{23}]$, C_2b_ = $(\bar{1}\bar{1}{\rm{22}})[\mathrm{11}\bar{2}3]$, C_3a_ = $(2\bar{1}\bar{1}2)[\bar{2}\mathrm{113}]$, C_3b_ = $(\bar{2}{\rm{112}})[2\bar{1}\bar{1}3]$, T_1a_ = $(1\bar{1}{\rm{02}})[\bar{1}\mathrm{101}]$, T_1b_ = $(\bar{1}{\rm{102}})[1\bar{1}\mathrm{01}]$, T_2a_ = $(\bar{1}{\rm{012}})[\mathrm{10}\bar{1}1]$, T_2b_ = $({\rm{10}}\bar{1}2)[\bar{1}\mathrm{011}]$, T_3a_ = $({\rm{01}}\bar{1}2)[0\bar{1}\mathrm{11}]$, and T_3b_ = $(0\bar{1}{\rm{12}})[\mathrm{01}\bar{1}1]$. The result shows that three primary $\{11\bar{2}2\}$ twin variants, C_1a_, C_2a_, and C_3a_, have a high SF of ~0.4 and they are all active, indicating that the variant selection during the primary $\{11\bar{2}2\}$ twinning is governed by the Schmid law. For the secondary $\{10\bar{1}2\}$ twinning, two variants T_3a_ and T_3b_ in primary $\{11\bar{2}2\}$ variant C_1a_, four variants T_1a_, T_1b_, T_3a_, and T_3b_ in primary $\{11\bar{2}2\}$ variant C_2a_, and two variants T_3a_ and T_3b_ in primary $\{11\bar{2}2\}$ variant C_3a_ have a high SF of >~0.3, and only variant T_3a_ with the highest SF of 0.5 is active in primary $\{11\bar{2}2\}$ variant C_1a_, indicating that the Schmid law is still valid. Therefore, we can conclude that the variant selection during the primary $\{11\bar{2}2\}$ twinning and secondary $\{10\bar{1}2\}$ twinning obeys the Schmid law.

**Table 2:** SF analysis on the primary $\{11\bar{2}2\}$ twin variants (PTV) and secondary $\{10\bar{1}2\}$ twin variants (STV) of grain A (*φ*_1_ = 189°, *Φ* = 32°, *φ*_2_ = 54°), indicated in the inverse pole figure map of the second stage of the rolled state in Fig. 2b.

PTV	SF	STV	SF		
	Matrix		C_1a_^a^	C_2a_^a^	C_3a_^a^
C_1a_	0.395*	T_1a_	0.130	0.328	0.097
C_1b_	0.167	T_1b_	0.133	0.350	0.102
C_2a_	0.392*	T_2a_	0.115	0.011	0.141
C_2b_	0.006	T_2b_	0.118	0.010	0.148
C_3a_	0.382*	T_3a_	0.500*	0.400	0.498
C_3b_	0.225	T_3b_	0.494	0.357	0.486

^a^C_1a_ [*φ*_1_ = 235°, *Φ* = 88°, *φ*_2_ = 29°], C_2a_ [*φ*_1_ = 5°, *Φ* = 81.4°, *φ*_2_ = 2°], and C_3a_ [*φ*_1_ = 133°, *Φ* = 86°, *φ*_2_ = 33°].

*Active twin variant.

**Figure 4:**
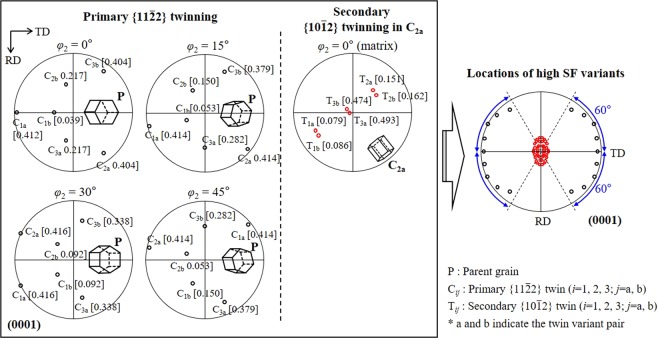
SF analysis on the primary $\{11\bar{2}2\}$ and secondary $\{10\bar{1}2\}$ twin systems under the rolling condition, i.e., the compression along the ND. Here, the parent grain is assumed to have a perfect TD split basal texture with a randomly oriented *a*-axis, i.e., *φ*_1_ = 180°, *Φ* = 30°, and 0° ≤ *φ*_2_ ≤60°, and the secondary $\{10\bar{1}2\}$ twinning is assumed to occur in primary twin variant C_2a_ (*φ*_1_ = 124°, *Φ* = 84°, *φ*_2_ = 35°). (0001) pole figure showing the locations of the high SF variants is presented in the right panel; the primary $\{11\bar{2}2\}$ twin variants with a SF of >~0.38 (black circle) and secondary $\{10\bar{1}2\}$ twin variants with a SF of >0.4 (red circle).

To obtain a more general insight on how the variant selection during the twinning affects the twinning-induced crystallographic lattice reorientation, we assumed a perfect TD split basal texture with the randomly oriented *a*-axis, i.e., *φ*_1_ = 0° or 180°, *Φ* = 30°, and 0° ≤ *φ*_2_ ≤ 60°, and conducted a SF analysis on the primary $\{11\bar{2}2\}$ and secondary $\{10\bar{1}2\}$ twin systems. Note that the SF values at both *φ*_2_ and 60°–*φ*_2_ are equal because of the unique angle relationship between the three *a*-axes of the HCP lattice. According to the result (Fig. 4), the three primary $\{11\bar{2}2\}$ twin variants from the three different twin variant pairs (two twin variants from two different twin variant pairs at *φ*_2_ = 30°) have a high SF of >~0.38, which varies with angle *φ*_2_. The physical meaning of *φ*_2_ is the rotation of the HCP lattice with respect to the *c*-axis so that it is related to the angle relationship between the *a*-axis and loading axis. The basal pole locations, predicted by the activation of such high SF variants (i.e., conforming to the Schmid law), are shown in the right panel of Fig. 4 and they are consistent with the texture component, with the *c*-axis distributed widely in the RD–TD plane, observed in the second stage of the rolled state. Because the secondary $\{10\bar{1}2\}$ twinning can occur in multiple primary $\{11\bar{2}2\}$ twin variants with a high SF of >~0.38, all the possible cases were considered in the analysis. The result for the specific case in which the secondary $\{10\bar{1}2\}$ twinning occurs in primary $\{11\bar{2}2\}$ twin variant C_2a_ (*φ*_1_ = 124°, *Φ* = 84°, *φ*_2_ = 35°) is presented in Fig. 4. Two $\{10\bar{1}2\}$ twin variants T_3a_ and T_3b_ from a single twin variant pair have a high SF of ~0.5 and their basal poles are located around the ND. Considering all the possible cases of the secondary $\{10\bar{1}2\}$ twinning in which the variants with a high SF of >0.4 are assumed to be active by obeying the Schmid law, the basal pole locations of the active secondary $\{10\bar{1}2\}$ twin variants are predicted to be located around the ND (the right panel of Fig. 4). These are in agreement with the texture component, with the *c*-axis aligned parallel to the ND, observed in the second stage of the rolled state. This agreement in the crystallographic textures from the predictions and experiments clearly supports that the Schmid law governs the variant selection during the primary $\{11\bar{2}2\}$ twinning and secondary $\{10\bar{1}2\}$ twinning, thereby controlling the twinning-induced crystallographic texture modification. The result further confirms that the two texture components with the *c*-axis distributed widely in the RD–TD plane and with the *c*-axis aligned parallel to the ND (hereafter referred to as the primary $\{11\bar{2}2\}$ twin texture and secondary $\{10\bar{1}2\}$ twin texture, respectively) originate from the primary $\{11\bar{2}2\}$ twinning and secondary $\{10\bar{1}2\}$ twinning, respectively. It is noted that the $\{10\bar{1}2\}$ twin is generated in the preformed $\{11\bar{2}2\}$ twin, consuming the $\{11\bar{2}2\}$ twin, so that with increasing thickness reduction in the second stage of the rolling, the primary $\{11\bar{2}2\}$ twin texture becomes weak whereas the secondary $\{10\bar{1}2\}$ twin texture intensifies (TCR #3 and #4).

### Mechanical properties and formability

The tensile properties and Lankford value *r*, defined as the ratio of the strains in the width and thickness directions, of the final sheets produced by the CCR and TCR processes are listed in Table 3. There is no relevant change in the ultimate tensile strength and elongation between the CCR and TCR processes. However, the yield strength is improved by the TCR process, and the improvement level depends on the loading direction (higher in the RD, whereas almost negligible in the TD) as well as the thickness reduction in the second stage of the rolling process. TCR #2 sheet offers the highest yield strength, which increases by 16% in the RD, 10% in the 45° direction, and 2% in the TD. The yield anisotropy is also reduced by the TCR process, and the lowest level is achieved for TCR #2 sheet. Lankford value *r* is significantly reduced in the 45° direction and TD, indicating that the thinning capability improves in both the directions, whereas it somewhat increases in the RD. It is known that the average *r*-value ($\bar{r}$ = [*r*_RD_ + 2*r*_45°_ + *r*_TD_]/4) and planar *r*-value (Δ*r* = (*r*_RD_ − 2*r*_45°_ + *r*_TD_)/4) are the measures of the thinning capability and planar (in-plane) anisotropy of a rolled sheet metal, respectively. Our result shows that the TCR process significantly increases the thinning capability and reduces the planar anisotropy of the CP-Ti sheets (Table 3). Maximum improvement is achieved in TCR #2 sheet with a 29% reduction in the average *r*-value and a 44% reduction in the planar *r*-value.

**Table 3:** Tensile properties and Lankford value *r* of the final sheets produced by the CCR and four designed TCR processes, and their loading direction dependence; yield strength (YS), ultimate tensile strength (UTS), and elongation (EL).

Rolling process	YS (MPa)			UTS (MPa)			EL (%)			r				
	RD	45°	TD	RD	45°	TD	RD	45°	TD	RD	45°	TD	$\bar{r}$ ^a^	Δr^b^
CCR	169	201	230	338	312	297	58.2	61.5	47.6	1.22	2.71	3.55	2.55	0.16
TCR #1	178	212	229	334	295	298	61.8	60.0	50.3	1.29	2.46	3.02	2.31	0.14
TCR #2	196	220	234	322	308	307	60.6	64.1	51.6	1.40	1.89	2.04	1.81	0.09
TCR #3	188	210	227	324	294	302	61.2	62.1	52.3	1.44	2.28	2.64	2.16	0.12
TCR #4	183	216	227	326	297	295	59.4	59.7	54.0	1.33	2.30	2.87	2.20	0.11

^a^Average *r*-value, $$\bar{r}$$ = (*r*_RD_ + 2*r*_45°_ + *r*_TD_)/4.

^b^Plana*r r*-value, Δ*r* = (*r*_RD_ − 2*r*_45°_ + *r*_TD_)/4.

**Figure 5:**
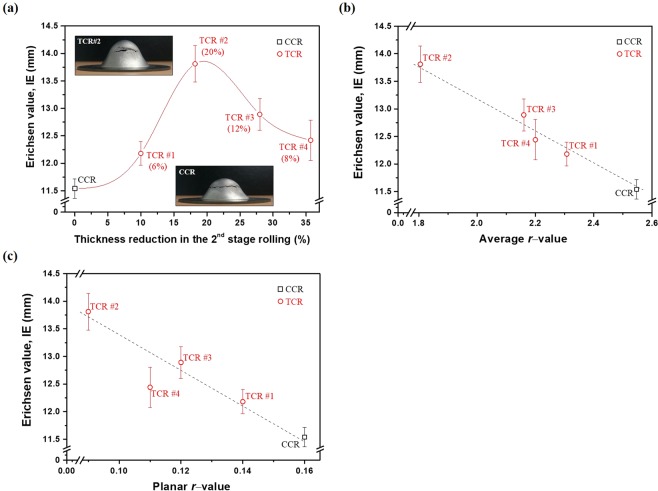
Comparison in the formability of the final sheets produced by the CCR and TCR processes. (**a**) Erichsen value as a function of thickness reduction in the second stage of the rolling process; the numbers in parentheses indicate the improvement of the formability with respect to the CCR. Erichsen value as a function of (**b**) average *r*-value and (**c**) planar *r*-value. Error bar indicates the standard deviation.

Figure 5 compares the formability (Erichsen value, IE) of the CCR and TCR sheets. The formability is evidently improved by the TCR process, but the improvement level is not in proportion to the thickness reduction in the second stage of the rolling (Fig. 5a). It increases with increasing thickness reduction, reaching a maximum improvement of 20% for TCR #2, but decreases with further increasing thickness reduction (TCR #3 and 4). This implies that the thickness reduction in the second stage of the rolling is not a decisive factor dominating the formability improvement. When the formability is plotted as a function of the average *r*-value (Fig. 5b) or planar *r*-value (Fig. 5c), a negative linear relationship is exhibited, indicating that the formability is closely associated with the thinning capability and planar anisotropy. A high thinning capability (low average *r*-value) and low planar anisotropy (low planar *r*-value) are beneficial for the formability improvement.

These results clearly support that the TCR process is able to improve the yield strength and formability simultaneously, and the improvement level is governed by two factors, the thinning capability and planar anisotropy, which are closely related to the thickness reduction in the second stage of the rolling. Considering that ASTM grade 1 CP-Ti generally has ~30% higher formability^25^ and ~60% lower yield strength^1^ than ASTM grade 2 CP-Ti (the material used in the present study), the 20% formability improvement of TCR #2 sheet suggests a possibility of replacing ASTM grade 1 CP-Ti with ASTM grade 2 CP-Ti in various industrial applications including heat exchangers (note that more improvement in the formability can be expected by optimizing the thickness reduction in the second stage of the rolling). In addition, the 16% enhanced yield strength in TCR #2 further allows the use of thinner sheets, saving Ti raw material and improving the heat transfer efficiency. Moreover, the suggested TCR process can be implemented simply by employing intermediate annealing at a late stage of the CCR process so that it can be easily deployed on a commercial scale. These advantageous features of the TCR process strengthen the competitiveness of the CP-Ti sheets in terms of the improved performance as well as low manufacturing cost.

### Mechanisms for improved yield strength and formability

As described earlier, the improvement of the yield strength and formability by the TCR process is attributed to the crystallographic texture modification caused by the formation of deformation twins in the second stage of the rolling. The change in the crystallographic lattice orientation will alter the activities of the plastic deformation mechanisms, consequently affecting the deformation behavior, mechanical properties, and formability. We analyzed the activities of four important deformation mechanisms, prismatic <*a*> slip, basal <*a*> slip, $\{11\bar{2}2\}$ twinning, and $\{10\bar{1}2\}$ twinning, by examining their activation stresses in the three texture components, the TD split basal texture, primary $\{11\bar{2}2\}$ twin texture, and secondary $\{10\bar{1}2\}$ twin texture. In the analysis, the perfect TD split basal texture with the randomly oriented *a*-axis (*φ*_1_ = 0° or 180°, *Φ* = 30°, and 0° ≤ *φ*_2_ ≤ 60°) together with the primary $\{11\bar{2}2\}$ and secondary $\{10\bar{1}2\}$ twin textures, predicted by obeying the SF law, were used for simplicity. The primary $\{11\bar{2}2\}$ twin texture with the *c*-axis aligned parallel to the RD–TD plane and distributed within ±60° from the TD toward the RD and the secondary $\{10\bar{1}2\}$ twin texture with the *c*-axis aligned parallel to the ND (Fig. 4) were considered. A SF analysis was first conducted and then the activation stress for each deformation mode (i.e., stress required to activate each deformation mode) was calculated by combining the SF and critical resolved shear stress (CRSS) (=CRSS/SF). The CRSSs used in the calculation were 78 MPa for prismatic <*a*> slip^26^, 125 MPa for basal <*a*> slip^26^, 186 MPa for $\{11\bar{2}2\}$ twinning^27^, and 99 MPa for $\{10\bar{1}2\}$ twinning^28^.

**Figure 6:**
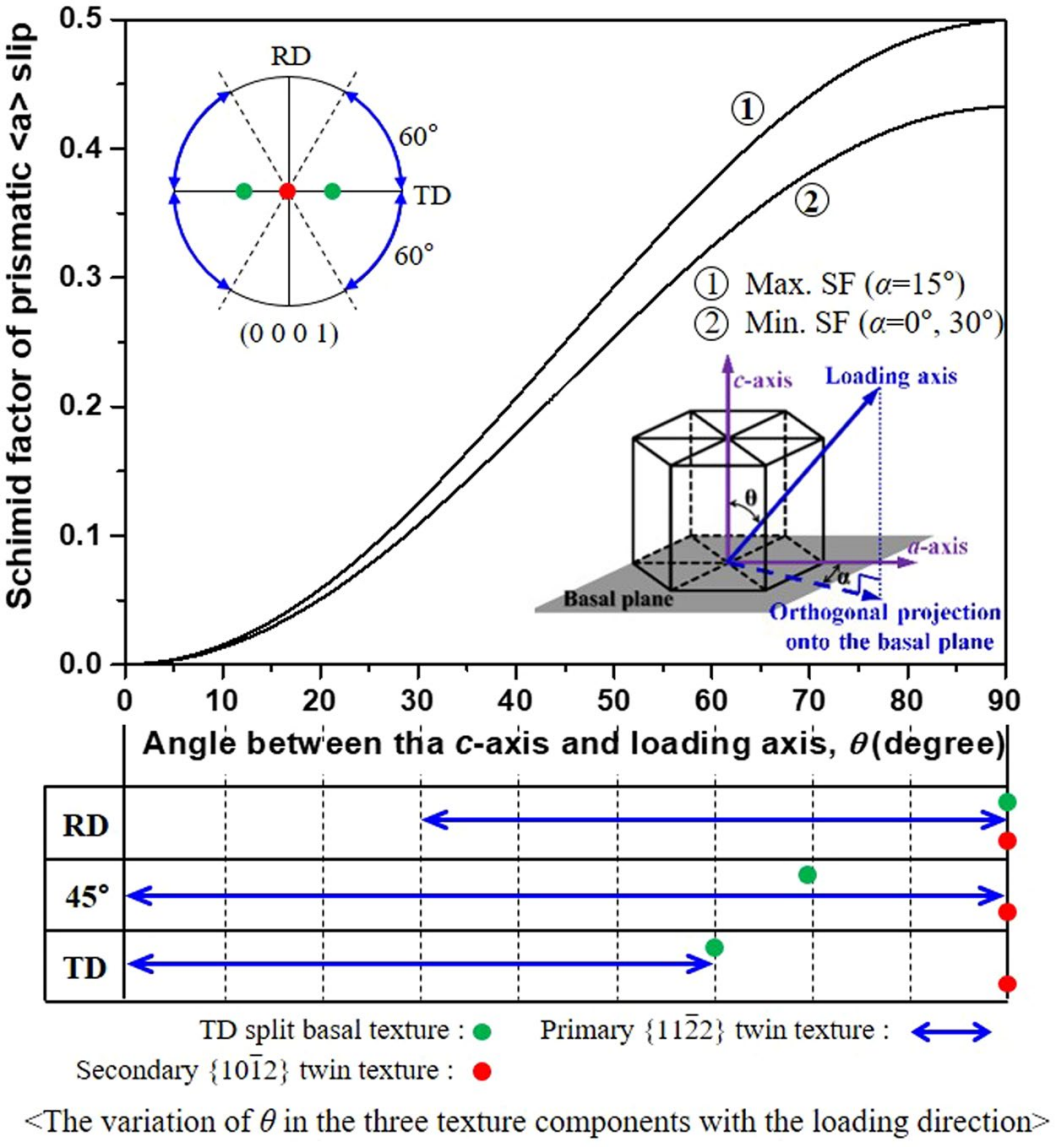
Maximum and minimum SF values of the prismatic <*a*> slip as a function of *θ*; the variation of *θ* in the three texture components with the three loading directions, RD, 45° direction and TD, is presented in the bottom panel. Here, *θ* is the angle between the *c*-axis and loading axis, and *α* is the angle between the *a*-axis and loading axis under the condition that the loading axis is projected onto the basal plane (refer to the inset).

According to previous studies^20,26^, prismatic <*a*> slip dominates the in-plane yielding of a rolled CP-Ti, and the variation in its SF with the loading direction results in a yield strength anisotropy. Therefore, the improvement in the yield strength by the TCR process can be understood by examining how the twinning-induced crystallographic lattice reorientation influences the SF of the prismatic <*a*> slip. Figure 6 shows the variation in the SF of the prismatic <*a*> slip with the loading direction in the three texture components; for the convenience of understanding, three representative loading directions, RD, 45° direction, and TD, are exemplified. The SF is mainly determined by angle *θ* between the *c*-axis and loading axis, and at a given *θ*, it also slightly varies with angle *α* between the *a*-axis and loading axis under the condition that the loading axis is projected onto the basal plane (refer to the inset of Fig. 6). For the TD split basal texture, *θ* ranges from 60° (TD) to 90° (RD), and the SF gradually increases with increasing *θ*, having a minimum of ~0.33 in the TD (*θ* = 60°, *α* = 0°, 30°) and a maximum of 0.5 in the RD (*θ* = 90°, *α* = 15°). These high SF values are indicative of the importance of the prismatic <*a*> slip in the deformation, supporting that the yield of the CCR sheet is dominated by the prismatic <*a*> slip. The relatively low SF values in the TD and 45° direction, compared to the RD, will increase the yield strength in both the directions, causing a yielding anisotropy, and this corresponds with the experimental result (Table 3). For the primary $\{11\bar{2}2\}$ twin texture, *θ* is widely distributed, ranging from 0° to 90°, and low SF values of <0.3 can be easily achieved at *θ* values of <~50°, making the activation of the prismatic <*a*> slip difficult. Such a hard crystallographic orientation for the prismatic <*a*> slip will increase the in-plane yield strength. For the secondary $\{10\bar{1}2\}$ twin texture, *θ* is typically 90° in any direction in the RD–TD plane, and this gives a SF of 0.5, facilitating the operation of the prismatic <*a*> slip and thereby decreasing the in-plane yield strength. Based on this result, we can speculate that to achieve the best improvement of the yield strength, the primary $\{11\bar{2}2\}$ twin texture should be maximized while suppressing the secondary $\{10\bar{1}2\}$ twin texture. This corresponds to TCR #2 in which the primary $\{11\bar{2}2\}$ twin texture is most intensified and the maximum yield strengths are achieved (Table 3). It is noted that the yield strength improvement is reduced for TCR #3 and #4, in which the primary $\{11\bar{2}2\}$ twin texture is weakened whereas the secondary $\{10\bar{1}2\}$ twin texture is intensified.

**Figure 7:**
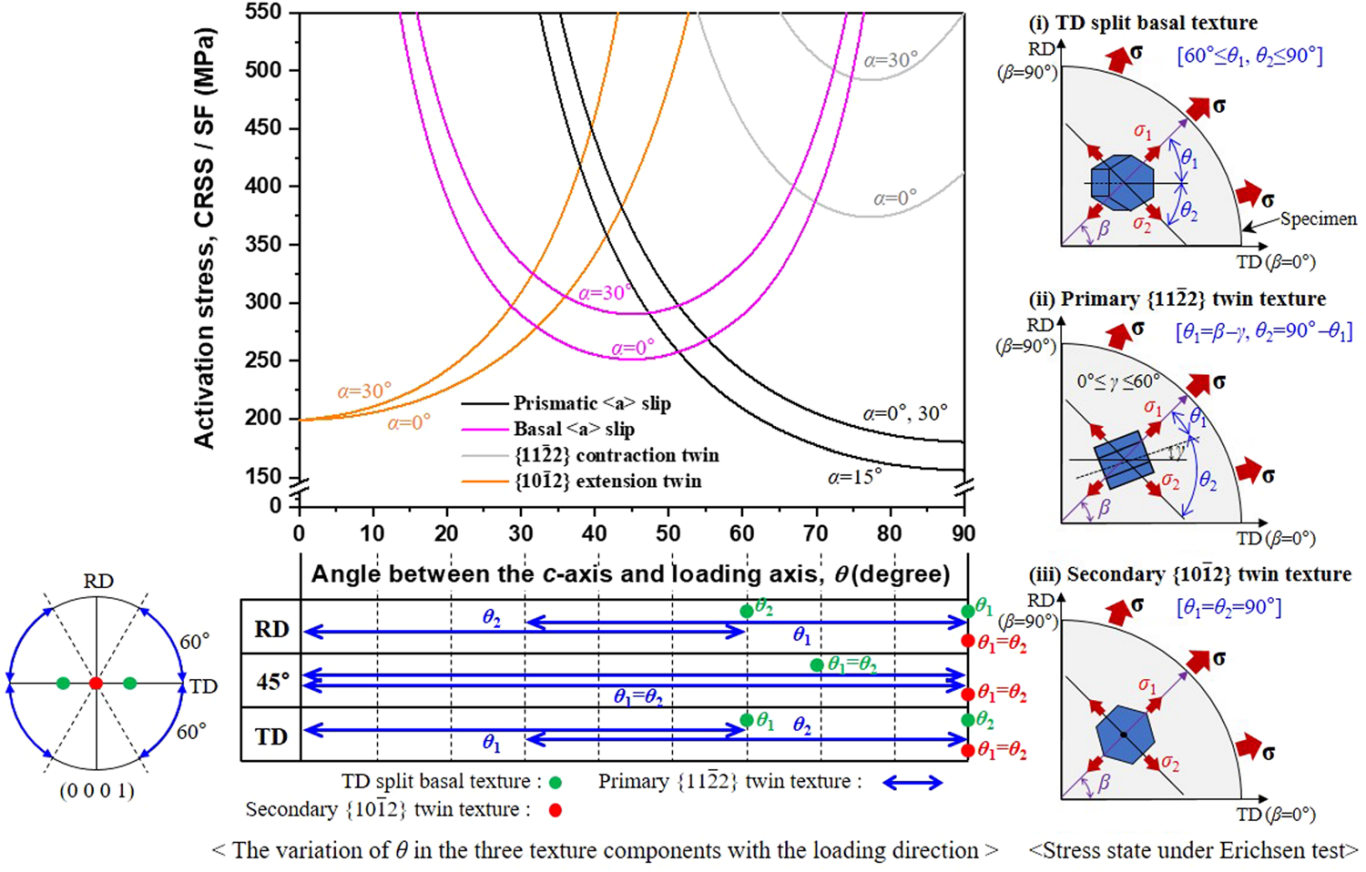
Activation stresses of the prismatic <*a*> slip, basal <*a*> slip, $\{11\bar{2}2\}$ contraction twinning, and $\{10\bar{1}2\}$ extension twinning as a function of *θ*. Here, *σ*_1_ is the radial stress, *θ*_1_ is the angle between the *c*-axis and *σ*_1_, *σ*_2_ is the circumferential stress, *θ*_2_ is the angle between the *c*-axis and *σ*_2_, *β* is the angle between *σ*_1_ and TD, and *γ* is the angle between the *c*-axis and TD.

The formability improvement by the TCR process was associated with the enhanced thinning capability and reduced planar anisotropy. Therefore, the responsible mechanisms for improving the formability can be understood by analyzing how the twinning-induced crystallographic lattice reorientation affects the thinning capability and planar anisotropy. Figure 7 displays the activation stresses of the four important plastic deformation mechanisms as a function of *θ*, where possible *θ* values in the three texture components are indicated in the three representative directions, RD, 45° direction, and TD. At a given *θ*, the deformation mode with the lowest activation stress is expected to be active and dominate the deformation. It is noted that because biaxial stresses with the same magnitude, radial stress *σ*_1_ and circumferential stress *σ*_2_, develop during the Erichsen test, the activation of each deformation mode is considered with these two stresses: *θ*_1_ and *θ*_2_ corresponding to *θ*, relating to *σ*_1_ and *σ*_2_, respectively. For the TD split basal texture, both *θ*_1_ and *θ*_2_ vary in the range of 60° to 90°, and the prismatic <*a*> slip has the lowest activation stress in this range, predicting its importance in the deformation. The activation stress of the prismatic <*a*> slip gradually decreases from 208 MPa (*θ*_1_ = 60° in the TD or *θ*_2_ = 60° in the RD) to 155 MPa (*θ*_1_ = 90° in the RD or *θ*_2_ = 90° in the TD) with increasing *θ*. However, in terms of the thinning capability, the prismatic <*a*> slip is not effective for accommodating a through-thickness strain because the $< 11\bar{2}0 >$ slip direction has a high angle relation of 60°–90° with the thickness direction (i.e., ND). Therefore, its contribution is mostly confined to the in-plane deformation. Such a deficiency in the deformation mechanisms that are able to accommodate the through-thickness strain reduces the thinning capability and consequently leads to a low formability, as evidenced in the CCR sheet. For the primary $\{11\bar{2}2\}$ twin texture, *θ* (*θ*_1_ and/or *θ*_2_) is widely distributed covering a broad range of 0°–90° so that three different deformation modes, $\{10\bar{1}2\}$ twinning, basal <*a*> slip, and prismatic <*a*> slip, can be active concurrently in any direction in the RD–TD plane. It is predicted that the $\{10\bar{1}2\}$ twinning will be active for 0° ≤ *θ* ≤ ~32°, basal <*a*> slip for ~32° ≤ *θ* ≤ ~51°, and prismatic <*a*> slip for ~51° ≤ *θ* ≤ 90°. Therefore, the combined effects of these three competing deformation mechanisms are expected to dominate the deformation behavior during the Erichsen test. The interesting point here is that unlike the case of the TD split basal texture, both the basal <*a*> slip and prismatic <*a*> slip can accommodate a through-thickness strain effectively, thereby enhancing the thinning capability, as the $< 11\bar{2}0 >$ slip direction makes a low angle relation of 0°–30° with the thickness direction. In addition, when the $\{10\bar{1}2\}$ twinning occurs, the HCP lattice extends along the *c*-axis and concurrently contracts in the direction perpendicular to the *c*-axis^29^. Thus, the $\{10\bar{1}2\}$ twinning can accommodate a through-thickness strain as well as an in-plane strain. However, as maximum achievable twinning strain *ε*_twin_ (the strain accommodation by $\{10\bar{1}2\}$ twinning) is estimated at less than ~5%, its contribution to the thinning capability improvement appears to be limited. ${\varepsilon }_{twin}=(1/\sqrt{2})\cdot {\gamma }_{twin}\cdot {f}_{twin}$^30,31^, where *γ*_twin_ is the characteristic twinning shear (0.176 for $\{10\bar{1}2\}$ twin^24^) and *f*_twin_ is the volume fraction of the $\{10\bar{1}2\}$ twin (the maximum achievable volume fraction of the $\{11\bar{2}2\}$ twin is less than ~40%^32^). Moreover, the wide distribution of the *c*-axis in the RD–TD plane improves the in-plane crystallographic symmetry, and thus, is beneficial for reducing the planar anisotropy. For the secondary $\{10\bar{1}2\}$ twin texture, both *θ*_1_ and *θ*_2_ are typically 90° in any direction in the RD–TD plane, and only the prismatic <*a*> slip is predicted to be active. Similar to the case of the TD split basal texture, the prismatic <*a*> slip is not effective for accommodating a through-thickness strain and its contribution is confined to the in-plane deformation, causing a low thinning capability and consequently leading to a poor formability because the $< 11\bar{2}0 >$ slip direction is perpendicular to the sheet thickness direction. However, the in-plane symmetric nature of the crystallographic orientation is expected to be beneficial for reducing the planar anisotropy. Based on these results, we attribute the formability improvement by the TCR process to the combined effects of the enhanced thinning capability through the operation of the prismatic <*a*> slip, basal <*a*> slip, and $\{10\bar{1}2\}$ twinning and the reduced planar anisotropy caused by the in-plane symmetric nature of the crystallographic orientation in the primary $\{11\bar{2}2\}$ twin texture. This is consistent with the experimental results where TCR #2 sheet with the most intense $\{11\bar{2}2\}$ twin texture exhibits the maximum improvement of the formability.

Our results reveal that the primary $\{11\bar{2}2\}$ twin texture is advantageous for the improvement of the yield strength and formability, whereas the secondary $\{10\bar{1}2\}$ twin texture impairs both the properties. To achieve the best improvement in both the properties, it is, therefore, suggested that the primary $\{11\bar{2}2\}$ twin texture be maximized while suppressing the secondary $\{10\bar{1}2\}$ twin texture. We can realize this by optimizing the thickness reduction in the second stage of the rolling, which is a major factor controlling the volume fractions (intensities) of the primary $\{11\bar{2}2\}$ twin and secondary $\{10\bar{1}2\}$ twin. Given that twinning activity is also affected by other factors including grain size, temperature, and deformation rate, the rolling parameters such as intermediate annealing temperature and time, rolling temperature, and rolling speed should be optimized^33–35^. It is noted that the optimizing process should be carefully conducted by considering the following two aspects: (1) the intricate relation between the volume fractions of the primary $\{11\bar{2}2\}$ twin and secondary $\{10\bar{1}2\}$ twin because the latter is generated in the former, thereby consuming it. (2) the crystallographic textures developed in the second stage of the rolled state are slightly changed by the intermediate annealing, although the main features remain almost unchanged (Fig. 2).

## Conclusions

In this study, a TCR process with intermediate annealing at a late stage of the CCR process was developed to control the crystallographic texture of a CP-Ti sheet. The intermediate annealing restored the twinning activity by eliminating the internal reaction stress developed in the first stage of the rolling through recrystallization. Consequently, the primary $\{11\bar{2}2\}$ twin and secondary $\{10\bar{1}2\}$ twin could be generated in the second stage of the rolling, which weakened and dispersed the TD split basal texture effectively. The crystallographic orientation of the primary $\{11\bar{2}2\}$ twin texture was found to be effective for the basal <*a*> slip, prismatic <*a*> slip, and $\{10\bar{1}2\}$ twinning to accommodate a through-thickness strain, thereby improving the thinning capability, as well as for reducing the planar anisotropy due to the in-plane crystallographic symmetry. These combined effects of the enhanced thinning capability and reduced planar anisotropy in the primary $\{11\bar{2}2\}$ twin texture led to an improvement of the formability. In terms of the yield strength, the primary $\{11\bar{2}2\}$ twin texture was barely oriented for the prismatic <*a*> slip, decreasing the SF and thereby increasing the in-plane yield strength. Therefore, the best improvement of both the yield strength and formability was achieved by maximizing the primary $\{11\bar{2}2\}$ twin texture while suppressing the secondary $\{10\bar{1}2\}$ twin texture. We demonstrated the feasibility of the developed two-stage cold rolling process with ASTM grade 2 CP-Ti, which showed a marked formability improvement by 20% comparable to the formability of ASTM grade 1 CP-Ti and a 16% yield strength improvement.

## Methods

### Material

The material used in this study was a hot-rolled and mill-annealed polycrystalline CP-Ti plate with 18 mm thickness. The interstitial solute composition was O–0.20, C–0.007, N–0.006, H–0.004, and Fe−0.04 (wt%), corresponding to ASTM grade 2 CP-Ti level. It had a twin-free equiaxed grain structure with an average grain size of 32 μm and a TD split basal texture with most of the *c*-axes inclined at angles of ±(25°–35°) from the ND toward the TD and with a randomly oriented *a*-axis in terms of the rotation of the HCP lattice with respect to the *c*-axis (Figure S1 in the Supplementary Information).

### Tensile and formability tests

Quasi-static uniaxial tensile tests were conducted along the featured three directions, i.e., RD, 45° direction in the RD–TD plane, and TD, by using an Instron 8801 testing machine (Instron, USA) at room temperature and a strain rate of 10^−3^ s^−1^ to measure the mechanical properties of the CP-Ti sheets produced by the CCR and TCR processes. Dogbone-shaped specimens with a gauge section of 25 mm × 6 mm × 1.8 mm (length × width × thickness) were machined from the CP-Ti sheets, in which the length directions (i.e., the loading axes) were aligned parallel to the RD, 45° direction in the RD–TD plane, and TD, respectively. Tensile tests of some of the specimens were interrupted at a strain of 12% to measure Lankford value *r* (=*ε*_width_/*ε*_thickness_). To measure the formability, the CP-Ti sheets were machined into disc-shaped specimens with 50 mm diameter and 1.0 mm thickness, and Erichsen tests were conducted by using a hemispherical-shaped punch with 20 mm diameter at a punch speed of 10 mm/min. The Erichsen value (IE), formability index, is defined as the depth of the impression at the fracture initiation. For a reliable analysis, all the tests were repeated more than thrice under each test condition.

### Crystallographic characterization

For crystallographic characterization, EBSD measurements were performed inside a scanning electron microscope (Helios NanoLab^TM^ 600, FEI Co., USA) operating at an acceleration voltage of 30 kV and a step size of 1 μm. The specimens were taken from the central region of the CP-Ti sheets, and their surface was mechanically polished and then electropolished with a solution of 410 mL methanol, 245 mL 2-butoxy ethanol, and 40 mL HClO_4_ 60%) using LectroPol-5 (STRUERS, USA) at a voltage of 22 V for 22 s. The measurement area was ~1.6 mm^2^, including ~1050 grains, and EBSD data with a confidence index of >0.1 were analyzed via the Orientation Imaging Microscopy 7.0 software (Edax, Inc., USA).

## Supplementary information

Figure S1

## Data Availability

All data generated or analyzed during this study are included in this published article (and its Supplementary Information files).
