# Supplementary material for: Simultaneous Improvement in the Strength and Formability of Commercially Pure Titanium via Twinning-induced Crystallographic Texture Control
Source: Sci Rep. 2019 Feb 14;9:2009. doi: 10.1038/s41598-019-38652-1 (PMC6376165; doi:10.1038/s41598-019-38652-1)
Supplement: Supplementary file 1 — Figure S1 [file 41598_2019_38652_MOESM1_ESM.docx]

Supplementary Information

Simultaneous Improvement in the Strength and Formability of Commercially Pure Titanium via Twinning-induced Crystallographic Texture Control

Jong Woo Won ^1^, Chan Hee Park ^1^, Jaekeun Hong ^1^, Chong Soo Lee ^2^, Seong-Gu Hong ^3, 4,^ *

^1^ Metal Materials Division, Korea Institute of Materials Science, Changwon 51508, Republic of Korea.

^2^ Graduate Institute of Ferrous Technology, Pohang University of Science and Technology, Pohang 37673, Republic of Korea.

^3^ Division of Industrial Metrology, Korea Research Institute of Standards and Science, Daejeon 34113, Republic of Korea.

^4^ Department of Nano Science, University of Science and Technology, Daejeon 34113, Republic of Korea.

* Corresponding author:

Seong-Gu Hong

Tel.: +82-42-868-5868, E-mail: [sghong@kriss.re.kr](mailto:sghong@kriss.re.kr)


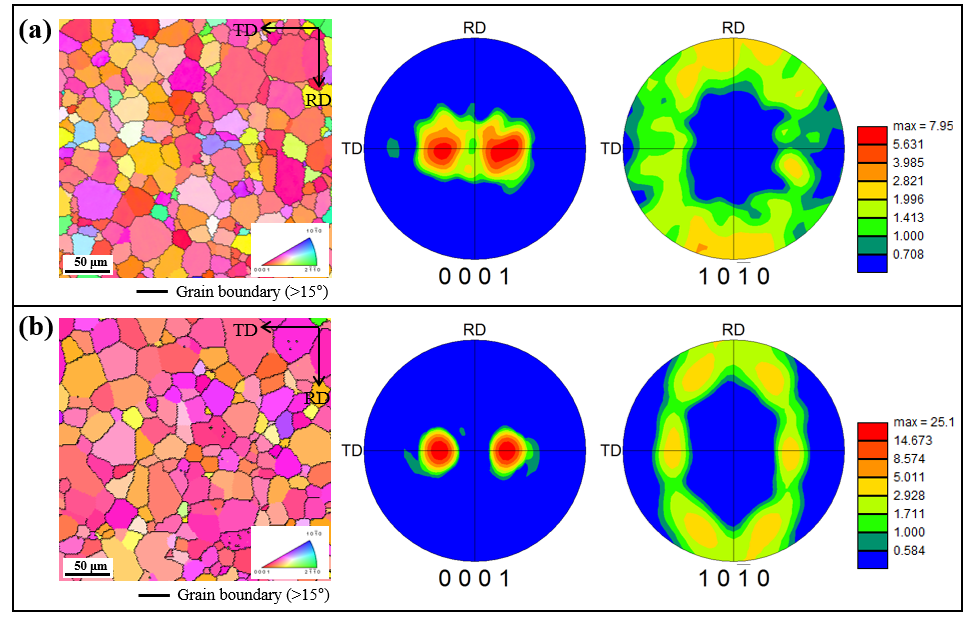


**Fig. S1.** Inverse pole figure maps, and (0001) and $\text{(10}\bar{\text{1}}\text{0)}$ pole figures. (a) the initial material (the hot-rolled and mill-annealed CP-Ti plate). (b) the CP-Ti sheet produced by the CCR process.
